# Growth Hormone Signaling in Bladder Cancer: Transcriptomic Profiling of Patient Samples and In Vitro Evidence of Therapy Resistance via ABC Transporters and EMT Activation

**DOI:** 10.3390/ijms26157113

**Published:** 2025-07-23

**Authors:** Emily Davis, Lydia J. Caggiano, Hannah Munholland, Reetobrata Basu, Darlene E. Berryman, John J. Kopchick

**Affiliations:** 1 Institute for Molecular Medicine and Aging, Heritage College of Osteopathic Medicine, Ohio University, Athens, OH 45701, USA; 2 Molecular and Cellular Biology Program, College of Arts and Sciences, Ohio University, Athens, OH 45701, USA; 3 Department of Biological Sciences, College of Arts and Sciences, Ohio University, Athens, OH 45701, USA; 4 Honors Tutorial College, Ohio University, Athens, OH 45701, USA; 5 Department of Biomedical Sciences, Heritage College of Osteopathic Medicine, Ohio University, Athens, OH 45701, USA

**Keywords:** growth hormone, growth hormone receptor, bladder cancer, therapy resistance, TCGA

## Abstract

Growth hormone (GH) signaling has been implicated in tumor progression and therapy resistance across multiple cancer types, yet its role in bladder cancer remains largely unexplored. In this study, we investigated the impact of GH and its receptor (GHR) on therapy resistance and disease progression in urothelial carcinoma (UC) through integrated transcriptomic and in vitro analyses. Transcriptomic profiling of The Cancer Genome Atlas bladder cancer cohort revealed that high tumoral *GHR* expression was associated with differential upregulation of genes involved in drug efflux, epithelial-to-mesenchymal transition (EMT), and extracellular matrix (ECM) remodeling. Notably, elevated *GHR* levels correlated with significantly reduced overall survival in patients with UC. In parallel, in vitro experiments demonstrated that GH promotes chemoresistance in UC cell lines via upregulation of ATP-binding cassette-containing (ABC) transporters and activation of EMT. GH also modulated ECM-remodeling-associated genes in a chemotherapy-dependent manner, including matrix metalloproteinases and tissue inhibitors of metalloproteinases. Importantly, these effects were abrogated by Pegvisomant, a GHR antagonist, indicating the functional relevance of GH/GHR signaling in the mediation of these phenotypes. Collectively, our findings support a mechanistic role for GH signaling in driving therapy resistance and tumor aggressiveness in bladder cancer and suggest GHR antagonism as a potential therapeutic strategy to improve treatment outcomes.

## 1. Introduction

Growth hormone (GH) is a peptide hormone secreted centrally from the anterior pituitary gland, which regulates longitudinal growth, organ development, and whole-body metabolism and promotes diseases such as diabetes and cancer. Additionally, GH is produced peripherally by many tissues, including tumors [1,2,3]. Individuals with acromegaly, characterized by excess GH secretion, have an increased risk for cancer [4,5,6], while individuals with Laron Syndrome (LS), characterized by resistance to GH, are protected against cancer [7,8,9]. Remarkably, in over 30 years of studying these individuals, only a single case of cancer has been documented [8]. This phenomenon is mirrored in mouse models. For example, mice that are transgenic for GH have increased incidence of cancer and tumor burden [10], while mice with an inactivating disruption in the GHR gene have decreased incidence of cancer and tumor burden [11].

GH has been linked to cancer development and progression since 1950 [12], with elevated levels of circulating GH reported in patients with cancer over the past 40 years [13,14]. Additionally, while high levels of GH and GHR expression are associated with poorer prognosis in several cancer types [15,16,17,18], this relationship is not consistent across all cancers—for example, it is not observed in liver cancer or renal clear cell carcinoma [19]. Nevertheless, evaluating this relationship can help to identify cancers that may benefit from treatment strategies that include targeting GH action.

Recent studies have begun to shed light on the specific molecular mechanisms by which GH contributes to cancer development and therapy resistance [15,17,19,20,21,22,23,24,25,26,27,28,29,30,31,32,33,34,35,36,37]. The role of GH in cancer therapy resistance involves facilitating active drug efflux, induction of phenotypic plasticity via epithelial-to-mesenchymal transition (EMT), modulation of the tumor microenvironment (TME), and enabling evasion of apoptosis [19,26,28,29,30,34,35,38,39]. Research performed by our laboratory has demonstrated that GH action upregulates expression of ATP-binding cassette-containing (ABC) transporters in cancer, contributing to heightened resistance to anticancer therapies in both in vitro and in vivo models [20,21,23,24,33]. Furthermore, our group and others have identified a critical role of GH in promoting EMT across multiple cancer types [17,18,24,27,34,37]. Although these GH-driven oncogenic processes have been extensively characterized in cancers of the breast, liver, colorectum, endometrium, and prostate, such studies have not been conducted in the context of bladder cancer [19]. Collectively, these findings highlight the potential of targeting GH action as a therapeutic strategy in cancer treatment.

Bladder cancer is one of the most common malignancies of the urinary system, with urothelial carcinoma (UC) comprising nearly 94% of cases. In 2025, an estimated 84,870 new cases are expected to be diagnosed in the United States [40]. While the five-year survival rate for early-stage bladder cancer is as high as 78.4%, it plummets to a mere 8.8% for patients with metastatic disease due to high recurrence rates and limited treatment efficacy [41]. The most common chemotherapeutic regimen for UC is a combination of cisplatin and gemcitabine, although cisplatin-based therapies reportedly have a high rate of failure [42,43]. The challenges in managing advanced UC stem from its tumoral heterogeneity, immune evasion, and resistance to standard therapies [44,45,46,47]. Advancing research into novel treatment strategies is critical for improving patient outcomes and long-term survival.

Previous research has implicated GH in bladder carcinogenesis in rats, with all tested animals developing UC [48]. More recently, *GHR* has been identified as a potential diagnostic marker for UC [49]. However, beyond these studies, the direct role of GH in UC remains largely unexplored. Downstream signaling molecules in the GH cascade, including STAT3/5 and MEK, have been associated with therapy resistance in UC [50,51], while insulin-like growth factor 1 (IGF-1), the primary downstream effector of GH, has also been linked to both therapy resistance and metastasis in UC [52,53]. Therapy resistance in UC arises from various mechanisms, such as evasion of apoptosis, drug efflux, metabolic reprogramming, and EMT [42,45,54,55,56,57]. Notably, many of these mechanisms intersect with those that are regulated by the action of GH [25].

In this study, we aimed to characterize the expression of *GHR* and its correlation with patient survival, therapy resistance, and disease progression in UC in silico. We used transcriptomic data from patients with UC in The Cancer Genome Atlas (TCGA) database to evaluate the expression level of *GHR* in UC tumors in comparison to noncancerous bladder tissue. To identify if bladder cancer may benefit from a treatment strategy that includes targeting GH action, we examined the relationship between *GHR* expression and patient survival. Additionally, we explored how *GHR* expression correlates with that of genes linked to therapy resistance and disease progression. We also aimed to begin the exploration of the role of GH in therapy resistance in UC. We first confirmed expression of *GHR* in UC cells and assessed their response to GH stimulation and its inhibition by Pegvisomant, an FDA-approved GHR antagonist, used for the treatment of acromegaly. Subsequently, we examined how GH and Pegvisomant affect the expression of drug efflux pumps, markers of EMT, matrix metalloproteinases (MMPs), and tissue inhibitors of metalloproteinases (TIMPs) in the presence and absence of the chemotherapeutic agents cisplatin and gemcitabine, which are standard treatments for UC [58,59,60]. Additionally, we assayed the effect of GH and Pegvisomant on cellular migration and invasion. Therefore, we sought to provide a foundational insight into the impact of GH on UC progression and therapy resistance, laying the groundwork for potential avenues for therapeutic intervention.

## 2. Results

### 2.1. GHR Is Expressed in UC In Silico and Correlates with Poor Overall Survival

**Figure 1:**
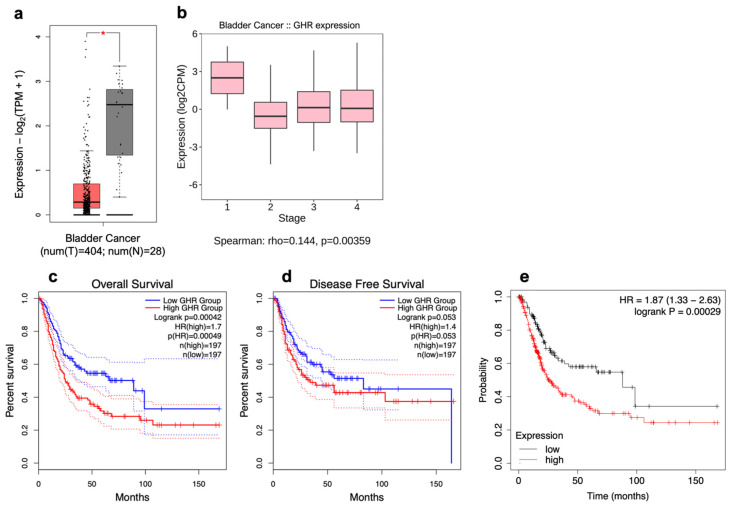
GHR is expressed in urothelial carcinoma. (**a**) Tumoral (left; red box) expression of *GHR* is significantly (* = *p* < 0.05) less than that in noncancerous urinary bladders (right; grey box) and (**b**) exhibits a weak but positive correlation with increasing clinical stages of UC. *GHR* expression is (**c**) significantly inversely correlated with overall survival (blue line = below median expression of *GHR*; red line = above median expression of *GHR*; dashed lines = 95% confidence interval) and (**d**) trends toward an inverse correlation with disease-free survival but does not reach statistical significance (blue line = below median expression of *GHR*; red line = above median expression of *GHR*; dashed lines = 95% confidence interval). (**e**) Tumoral expression of *GHR* and *PRLR* is significantly inversely correlated with patient survival (black line = below median expression of *GHR* and *PRLR*; red line = above median expression of *GHR* and *PRLR*).

Using transcriptomic data archived in TCGA obtained from 404 patients with bladder cancer [61], we found that, overall, *GHR* was not expressed as highly in bladder tumors as it was in normal bladder tissue taken from the same patients (Figure 1a). However, upon further analysis of the correlation between *GHR* expression and tumor stage, we found that GHR expression is significantly positively correlated with increasing tumor stage (rho = 0.144, *p* = 0.00359) (Figure 1b).

From transcriptomic data archived in TCGA database obtained from 394 patients with bladder cancer, a significant decrease in overall survival (hazard ratio (HR) = 1.7, *p* = 0.00042) and a trend toward a reduction in disease-free survival (HR = 1.5, *p* = 0.053) were observed in patients whose tumors express high (above median) levels of *GHR* in comparison with patients whose tumors express low (below median) levels of *GHR* (Figure 1c,d). While *GHR* levels may be lower in bladder tumors relative to normal tissue, higher expression of *GHR* within a tumor is still correlated with worse patient outcomes. This distinction highlights the importance of relative *GHR* expression specifically within cancerous tissue as a potential prognostic marker.

Often considered an extension of the GH gene family, the prolactin (*PRL*) gene has 83.4% sequence identity with human GH-N at the nucleotide level [62]. While the sequence identity of the *GHR* and the *PRL* receptor (*PRLR*) is much lower at only ~28%, their structures are extremely similar [63]. Uniquely, in humans, GH can bind to and activate PRLR in addition to its own cognate receptor. However, PRL can bind only to its own receptor [64]. Tumoral *GHR* and *PRLR* expression are significantly inversely correlated with the overall survival of patients with bladder cancer (HR = 1.87, *p* = 0.00029), as shown in Figure 1e.

For further validation, Kaplan–Meier survival plots with *GHR*, *GH1*, IGF-1 receptor (*IGF1R*), or *IGF1* as the reference gene were generated from TCGA database, including 405 patients with bladder cancer. Here, transcriptomic data from patients with bladder cancer were again parsed as above and below median mRNA expression levels, and correlation with overall survival was plotted with respect to sex, stage, and race.

**Figure 2:**
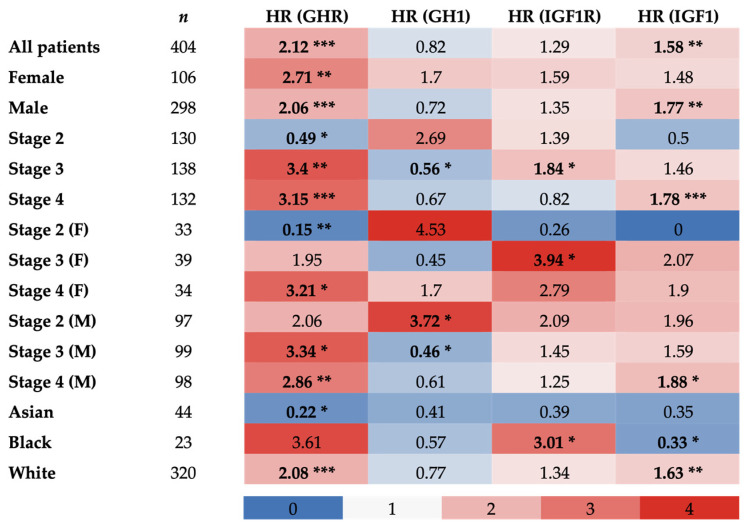
Hazard ratios of overall survival of patients with UC in TCGA database correlated with tumoral *GHR*, *GH1*, *IGF1R*, and *IGF1* expression. Bold values indicate a statistically significant correlation with survival (* = *p* < 0.05; ** = *p* < 0.01; *** = *p* < 0.001).

In both female and male sexes as well as the combination of both sexes, *GHR* expression was significantly inversely correlated with survival (respectively, HR = 2.71, 2.06, 2.12; *p* = 0.0071, 7.7 × 10^−5^, 2.3 × 10^−5^). *GHR* expression in clinical stage 4 was also inversely correlated with survival in female and male patients alone, as well as the combination of female and male patients (respectively, HR = 3.21, 2.86, 3.15; *p* = 0.0017, 0.019, 8.5 × 10^−5^). *GHR* expression in clinical tumor stage 3 was inversely correlated with survival in male patients alone and the combination of female and male patients (respectively, HR = 3.34, 3.40; *p* = 0.016, 0.0027). No significant correlation was observed between *GHR* expression in clinical stage 3 in female patients and survival. However, *GHR* expression in clinical stage 2 only trended toward an inverse correlation with survival in male patients but did not reach statistical significance (HR = 2.06, *p* = 0.058). *GHR* expression in clinical stage 2 in female patients alone and the combination of female and male patients was positively correlated with survival (respectively, HR = 0.15, 0.49; *p* = 0.0092, 0.035). Correlation between *GHR* expression in clinical stage 1 and survival was unable to be analyzed due to the insufficient number of patients for which data were available. Lastly, *GHR* expression in white patients was also significantly inversely correlated with survival (HR = 2.08; *p* = 2.7 × 10^−5^) and trended toward an inverse correlation in Black patients without reaching statistical significance (HR = 3.61, *p* = 0.077), while the opposite was true for Asian patients (HR = 0.22, *p* = 0.034) (Figure 2 and Figure S1).

In females, *GH1* expression trended toward an inverse correlation with survival (HR = 1.7; *p* = 0.083), while the opposite was true in males (HR = 0.72, *p* = 0.069), with neither reaching statistical significance, and no correlation was observed in the combination of both sexes (HR = 0.82; *p* = 0.2). *GH1* expression in clinical stage 2 in male patients was inversely correlated with survival, trended toward an inverse correlation in the combination of female and male patients without reaching statistical significance, and was not correlated with survival in female patients (respectively, HR = 3.72, 2.69, 4.53; *p* = 0.021, 0.053, 0.13). Contrarily, a positive correlation between *GH1* expression and survival was also observed in clinical stage 3 in male patients and the combination of female and male patients (respectively, HR = 0.46, 0.56; *p* = 0.025, 0.044), and a trend toward a positive correlation was observed in female patients but statistical significance was not reached (HR = 0.45; *p* = 0.09). While there were no significant correlations between *GH1* expression in clinical stage 4 for any patients and survival, both male patients and the combination of female and male patients trended toward a positive correlation (respectively, HR = 0.61, 0.67, *p* = 0.056, 0.084). Correlation between *GH1* expression in clinical stage 1 and survival was unable to be analyzed due to the insufficient number of patients for which data were available. Additionally, no significant correlation was observed between *GH1* expression and survival for Asian, Black, or white patients with UC (Figure 2 and Figure S2).

A significant inverse correlation between tumoral *IGF1R* expression and survival was observed in clinical stage 3 for female patients and the combination of female and male patients (respectively, HR = 3.94, 1.84; *p* = 0.019, 0.042). A trend toward an inverse correlation was observed in clinical stage 4 for female patients (HR = 2.79; *p* = 0.087) and clinical stage 2 for male patients (HR = 2.09; *p* = 0.057), while, conversely, a trend toward a positive correlation was observed between tumoral *IGF1R* in clinical stage 2 for female patients and survival (HR = 0.26; *p* = 0.077), but statistical significance was not reached in any case. Additionally, a significant inverse correlation was found between tumoral *IGF1R* expression and survival in Black patients (HR = 3.01; *p* = 0.047). No significant correlations were detected between tumoral *IGF1R* expression and survival in the following categories: all patients combined, female patients, male patients, clinical stages 2 and 4 for all patients, clinical stages 3 and 4 for male patients, Asian patients, and white patients (Figure 2 and Figure S3).

In both male patients and the combination of female and male patients, tumoral *IGF1* expression was significantly inversely correlated with survival (respectively, HR = 1.77, 1.58; *p* = 0.0023, 0.0025). This trend was also observed in clinical stage 4 for the same patients (respectively, HR = 1.88, 1.78; *p* = 0.026, 0.009). A trend toward an inverse correlation between *IGF1* and survival was present in clinical stage 2 for male patients (HR = 1.96; *p* = 0.074), whereas the opposite was true in clinical stage 2 for female patients (HR = 0; *p* = 0.058), but statistical significance was not reached in either case. Additionally, a significant inverse correlation between tumoral *IGF1* and survival was seen in white patients, with the opposite observed in Black patients (respectively, HR = 1.63, 0.33; *p* = 0.0029, 0.047). No significant correlations were detected between tumoral *IGF1* expression and survival in female patients, clinical stages 2 and 3 for all patients, clinical stages 3 and 4 for female patients, clinical stage 3 for male patients, and Asian patients (Figure 2 and Figure S4).

### 2.2. Genes Correlated with GHR Indicate a Therapy Resistance Gene Expression Pattern in UC

**Figure 3:**
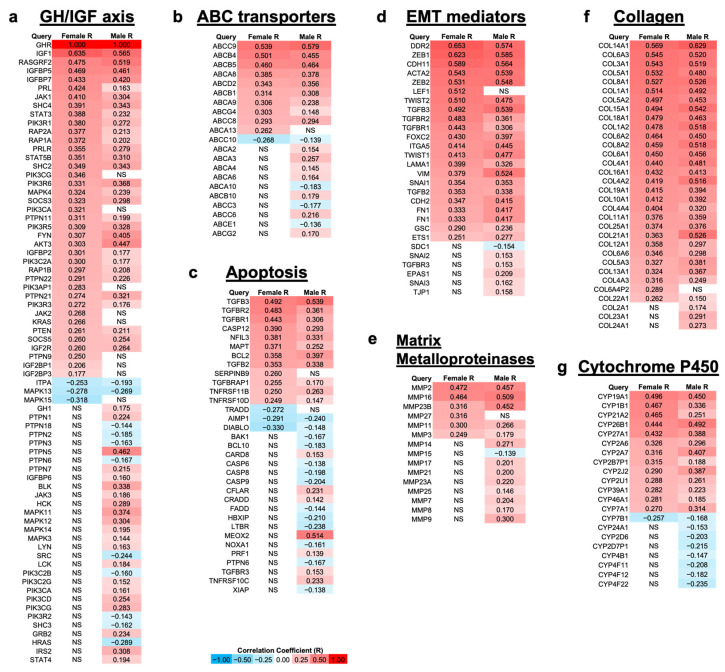
Genes involved in processes related to therapy resistance are differentially up- and downregulated in female (*n* = 108) and male (*n* = 304) patients with UC that have a relatively high mRNA expression level of *GHR* (FDR ≤ 0.05) via TCGA. (**a**) Genes involved with GH signaling; (**b**) ATP-binding cassette (ABC) transporters, which are nonspecific drug efflux pumps powered by ATP that have been shown to play a role in cancer drug resistance; (**c**) genes involved with apoptosis; (**d**) genes involved in epithelial-to-mesenchymal transition (EMT), which is the beginning of the metastatic process, and it occurs when cells lose epithelial morphology and gain mesenchymal morphology; (**e**) matrix metalloproteases, which create space for tumors to grow; (**f**) collagens; (**g**) cytochrome P450s. NS = not significantly correlated with *GHR* expression.

Using data from patients with bladder cancer in TCGA database, correlation analyses of differentially expressed mRNAs with respect to *GHR* mRNA expression in tumor samples were performed. Specific gene modules were queried on the basis of both their roles in driving therapy resistance and past reports of GH-regulated changes in their expression. In the GH–IGF axis module [65,66,67], significantly (False Detection Rate (FDR) ≤ 0.05) upregulated genes in both sexes included several genes known to be under the control of GH, such as *IGF1* and *IGF*-binding protein (IGFBP)-5, -7, and -2. We also observed significant upregulation in both sexes of downstream signaling molecules in the GH signaling cascade: *RASGRF*, Janus kinase (*JAK*)*1/2*, signal transducer and activator of transcription (*STAT*)*3/5*, phosphatidylinositol-3 kinase (*PI3K*), the SRC family kinase FYN, protein kinase B (*AKT*), and mitogen-activated protein kinase (*MAPK*). In addition, the upregulation in both sexes of suppressors of cytokine signaling (*SOCS3*, *SOCS5*, *PTPNs*) demonstrates active GH signaling in bladder cancer tumors (Figure 3a).

In the ABC transporters module [68,69,70], we observed significant upregulation of multidrug efflux pumps that have previously been reported to be upregulated by GH in other cancers [33]: *ABCB5* and *ABCB1* in both sexes and *ABCG2* in males only. In addition, many ABCA family transporters were significantly upregulated in male patients—*ABCA2*, *ABCA3*, *ABCA4*, and *ABCA6*—with *ABCA13* upregulated only in female patients and ABCA8 and ABCA9 significantly upregulated in both sexes (Figure 3b).

In the apoptosis module [71,72,73,74,75], multiple pro-apoptotic genes, such as Bcl-2 homologous antagonist killer (*BAK*), NADPH oxidase activator 1 (*NOXA1*), and caspase-6, -8, and -9, were significantly downregulated in male patients, as well as *DIABLO* in both sexes. This was accompanied by upregulation of anti-apoptotic genes, such as nuclear factor, interleukin-3 regulated (*NFIL3*), B-cell lymphoma 2 (*BCL2*), and tumor necrosis factor receptor superfamily (*TNFRSF*)-*11B* and -*10D* in both sexes (Figure 3c).

In the EMT mediators module [76,77,78], all genes, with the exception of syndecan 1 (*SDC1*), were significantly upregulated in at least one sex in patients with high GHR expression. The EMT-promoting transcription factors zinc finger E-box binding homeobox (*ZEB*)*1*, *ZEB2*, *TWIST1*, *TWIST2*, *SNAI1*, *FOXC2*, goosecoid (GSC), and *ETS1* were significantly upregulated in both sexes. *SNAI2* and *SNAI3* were upregulated in male patients, and *LEF1* was upregulated in female patients. Additionally, the mesenchymal markers cadherin (*CDH*)*2*, *CDH11*, smooth muscle α-actin 2 (*ACTA2*), α5β1 integrin (*ITGA5*), vimentin (VIM), and fibronectin (*FN1*) were all significantly upregulated in both sexes. Many genes related to transforming growth factor beta (TGF-β) signaling were significantly upregulated: *TGFB2*, *TGFB3*, *TGFBR1*, and *TGFBR2* in both sexes, with the addition of TGFBR3 in males (Figure 3d).

Similar to the EMT mediators module, in the MMPs module [79,80,81,82], all genes, with the exception of *MMP15*, were significantly upregulated in at least one sex. The MMPs with the highest correlation with GHR expression in both female and male patients with bladder cancer were *MMP2* (rho = 0.472 (F), 0.457 (M)), *MMP16* (rho = 0.464 (F), 0.509 (M)), and MMP23B (rho = 0.316 (F), 0.452 (M)) (Figure 3e).

Collagens are a major component of the extracellular matrix (ECM) that is digested by MMPs. Additionally, similar to both the EMT mediators and MMPs modules, all genes in the collagen module [83,84] were significantly upregulated with increasing GHR expression in at least one sex. Most of the collagens in this module are ECM constituent collagens, while only a few are constituents of the basement membrane (Figure 3f).

In the cytochrome P450 (CYP) module [85,86,87], the genes with the highest correlation with GHR expression were *CYP19A1* (rho = 0.496 (F), 0.450 (M)), *CYP1B1* (rho = 0.467 (F), 0.336 (M)), and *CYP21A2* (rho = 0.465 (F), 0.251 (M)). Many CYPs were also significantly inversely correlated with GHR expression in male patients with bladder cancer, and *CYP7B1* was downregulated with increased GHR expression in both sexes (Figure 3g).

### 2.3. GHR Is Expressed in UC Cells

**Figure 4:**
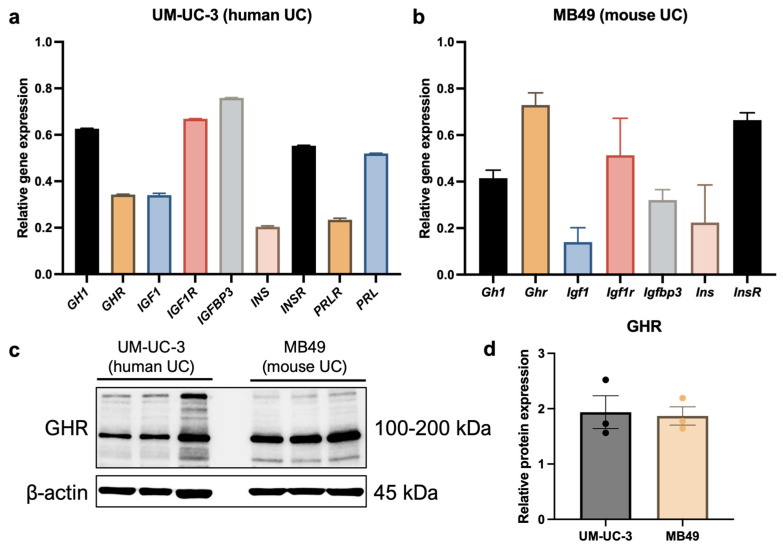
Human and mouse UC cells express GHR. (**a**) The human UC cell line UM-UC-3 expresses *GHR* mRNA, confirmed by qPCR. This cell line also exhibits relatively high expression of other genes involved in GH signaling, including *GH1*, *IGF1R*, and *IGFBP3.* (**b**) The murine UC cell line MB49 expresses *Ghr* mRNA, confirmed by qPCR. This cell line also exhibits relatively high levels of *Igf1r* and *Insr*. (**c**,**d**) High expression of GHR at the protein level was also confirmed by western blot in human and mouse UC cells.

To evaluate mRNA levels of genes involved in GH signaling, cells were starved in FBS-free growth media for 6 h. In human UC cells, *GH1*, *IGF1R*, and *IGFBP3* were highly expressed, while *GHR*, *IGF1*, *PRL*, and insulin receptor (*INSR*) were expressed at intermediate levels. In contrast, *PRLR* and insulin (*INS*) were expressed at relatively low levels (Figure 4a). In murine UC cells, *Ghr*, *Igf1r*, and *Insr* were highly expressed, whereas *Gh1*, *Igf1*, *Igfbp3*, and *Ins* exhibited low-to-intermediate expression (Figure 4b). Since GH only signals through the prolactin receptor in humans, mRNA expression levels of prolactin and its receptor were not evaluated in murine cells. Additionally, protein analysis confirmed high expression of GHR in both human and mouse UC cells (Figure 4c,d).

### 2.4. UC Cells Are Responsive to GH

**Figure 5:**
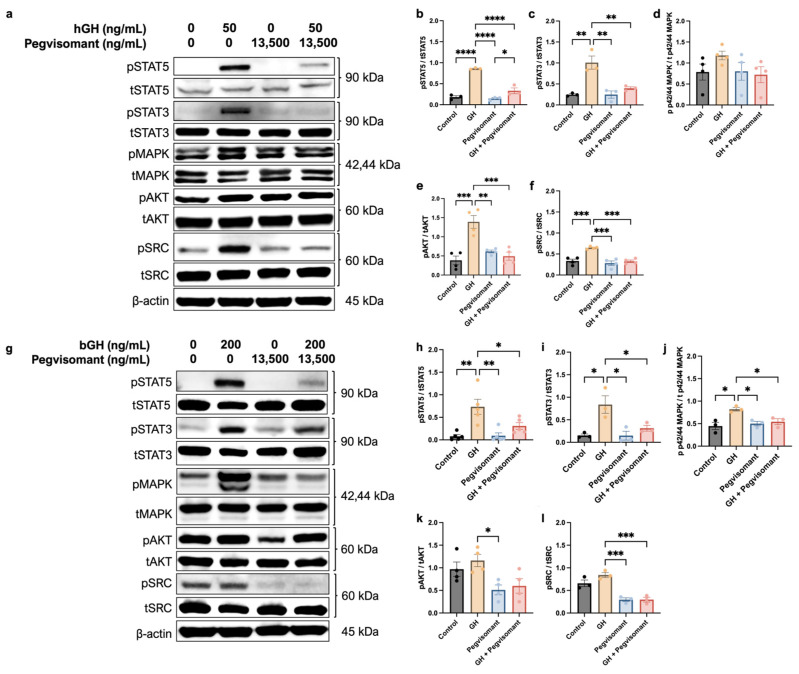
UC cells are responsive to GH. (**a**) Western blots show that GH stimulation to human UC cells increases phosphorylation of (**b**) STAT5, (**c**) STAT3, (**e**) AKT, and (**f**) SRC, which is reversed by the addition of Pegvisomant. (**d**) Phosphorylation of MAPK was unaffected by GH or Pegvisomant. (**g**) Western blots show that GH stimulation increases phosphorylation of (**h**) STAT5, (**i**) STAT3, and (**j**) MAPK, which is reversed by the addition of Pegvisomant. While GH did not increase phosphorylation of (**k**) AKT or (**l**) SRC, Pegvisomant decreased it. (* = *p* < 0.05; ** = *p* < 0.01; *** = *p* < 0.001 **** = *p* < 0.0001).

To assess the response of UC cells to GH and Pegvisomant, cells were serum-starved in FBS-free growth media for 6 h before being treated with GH and Pegvisomant for 12 min. Upon GH stimulation, human UC cells exhibited increased phosphorylation of STAT5, STAT3, AKT, and SRC. Treatment with Pegvisomant both alone and in combination with GH resulted in decreased phosphorylation of STAT5, STAT3, AKT, and SRC. However, p42/44 mitogen-activated protein kinase (MAPK) phosphorylation remained unaffected under all tested conditions, as expected due to the fact that UM-UC-3 cells are positive for the gain-of-function KRAS^G12C^ mutation (Figure 5a–f) [88].

Similarly, stimulation of mouse UC cells with GH resulted in increased phosphorylation of STAT5, STAT3, and MAPK, which was reversed when Pegvisomant was administered alone. Administration of Pegvisomant also decreased the phosphorylation of AKT and SRC. However, when GH and Pegvisomant were combined, MAPK phosphorylation remained elevated, indicating that Pegvisomant failed to inhibit this effect. When the combination of GH and Pegvisomant was administered, phosphorylation of STAT5, STAT3, and SRC was decreased (Figure 5g–l).

### 2.5. GHR Antagonism Decreases Expression of ABC Transporters in UC Cells

It is well known that GH regulates the expression and activity of ABC transporters in tumor cells [15,20,23,24,33]. ABC transporters function as multidrug efflux pumps, and they play a critical role in actively pumping out chemotherapeutic agents from cancer cells, thereby contributing to the development of chemoresistance [19,89,90]. Here, we examined the effect of GH, Pegvisomant, and the chemotherapies cisplatin and gemcitabine in various combinations on the protein levels of ABCB1, ABCC1, ABCC2, and ABCG2, as they have been reported to mediate GH-induced chemoresistance [20,33].

**Figure 6:**
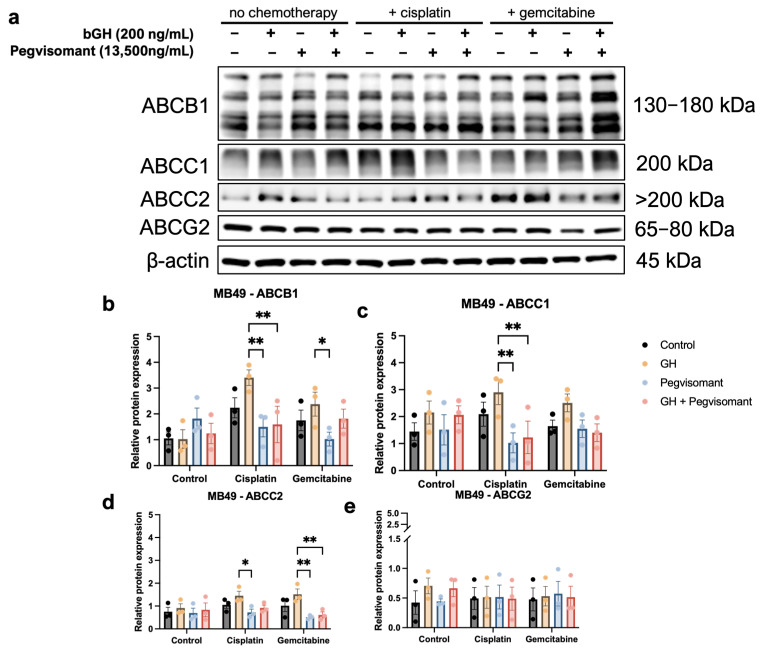
GH increases expression of ABC transporters in murine UC cells when treated with cisplatin or gemcitabine and GH. (**a**) Western blots and (**b**–**e**) quantification show increased expression of ABCB1, ABCC2, and ABCC2 in the presence of cisplatin and/or gemcitabine when exposed to GH. Pegvisomant is able to reverse this upregulation. (* = *p* < 0.05; ** = *p* < 0.01).

In mouse UC cells, ABC transporter expression remained unchanged in the absence of chemotherapeutic agents. Upon cisplatin treatment, GH stimulation significantly upregulated ABCB1 and ABCC1 expression compared with GH treatment alone. Pegvisomant successfully reversed this effect, both alone and in combination with GH (Figure 6a–c). Furthermore, Pegvisomant alone reduced expression of ABCB1 in the presence of gemcitabine (Figure 6a,b). ABCC2 expression was suppressed by Pegvisomant in gemcitabine-treated cells regardless of the presence of GH, whereas, in the presence of cisplatin, Pegvisomant failed to lower ABCC2 expression in GH-treated cells (Figure 6a,d). No significant changes were detected in ABCG2 expression under any condition (Figure 6a,e).

### 2.6. GHR Antagonism Inhibits Cellular Migration and Invasion in UC Cells

**Figure 7:**
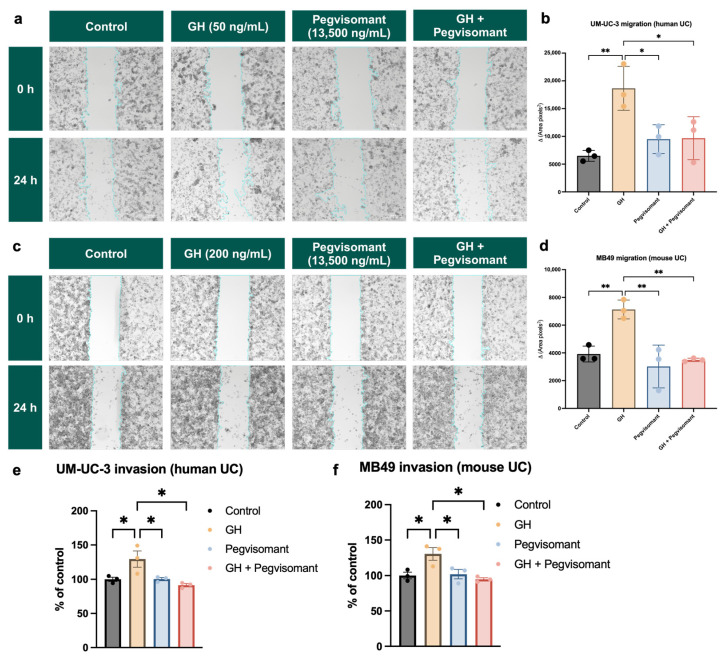
GHR antagonism inhibits cellular migration and invasion in UC cells. Wound-healing migration assay in (**a**,**b**) human and (**c**,**d**) mouse UC cells shows that GH treatment significantly reduces cell-free area, indicating increased cellular migration, while Pegvisomant reverses this effect. Fluorometric invasion assay in (**e**) human and (**f**) mouse UC cells demonstrates that GH stimulation increases basement membrane invasion, which is blocked by Pegvisomant. (* = *p* < 0.05; ** = *p* < 0.01).

GH action is a key regulator of tumor cell migration and invasion [28,36,91]. To assess the impact of GH action on the migratory potential of UC cells, we utilized a wound-healing migration assay [92]. Cells were treated with GH, Pegvisomant, or the combination of GH and Pegvisomant, and cellular migration was assessed by measuring the change in cell-free area before and after treatment. In human UC cells, stimulation with GH led to a significant reduction in cell-free area, demonstrating an increase in cellular migration. This effect was reversed when Pegvisomant was added to cells both alone and in combination with GH, suggesting an inhibitory role in GH-induced migration (Figure 7a,b). Likewise, in mouse UC cells, GH treatment promoted enhanced cellular migration, as evidenced by a decrease in cell-free area. This effect was effectively counteracted by Pegvisomant, both in the presence and absence of GH (Figure 7c,d).

Using a fluorometric assay, we evaluated the impact of GH and Pegvisomant on the basement membrane invasion capacity of UC cells. Stimulation with GH enhanced cellular invasion of human UC cells. This effect was reversed by Pegvisomant, both alone and in combination with GH (Figure 7e). A similar pattern was observed in murine UC cells, where GH stimulation led to a significant increase in basement membrane invasion capacity. This phenomenon was effectively reversed when Pegvisomant was administered, both in the presence and absence of GH, indicating that GH-driven invasion is inhibited by Pegvisomant (Figure 7f).

### 2.7. GH Modulates Expression of Markers of EMT and ECM Remodeling in UC Cells

One of the most well-characterized processes that drives cancer metastasis and is considered a key initiator of tumoral dissemination is EMT [78]. Previous research from our lab has identified GH as a key regulator of EMT both in vitro and in vivo [24,27,33]. Additionally, GH has been shown to upregulate expression of MMPs, which are potent pro-EMT factors that degrade the ECM, enabling tumor growth, invasion, and eventual metastasis [31,32,79,93]. To investigate the role of GH in EMT and ECM remodeling, we assessed protein expression levels of EMT markers, pro-EMT transcription factors, TGF-β, MMPs, and their natural inhibitors, TIMPs, under the previously described treatment conditions.

**Figure 8:**
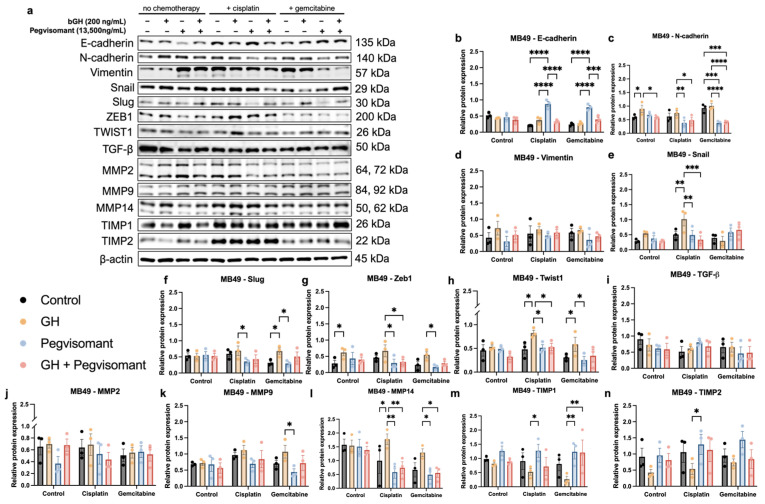
GH modulates expression of markers of EMT and ECM remodeling in mouse UC cells. (**a**) Western blots with (**b**–**n**) quantification. (* = *p* < 0.05; ** = *p* < 0.01; *** = *p* < 0.001; **** = *p* < 0.0001).

In mouse UC cells, we observed no significant changes in the expression levels of E-cadherin, Snail, Slug, Twist1, MMP9, MMP14, TIMP1, or TIMP2 in the absence of chemotherapy. However, expression of N-cadherin and Zeb1 was increased by the addition of GH. While Pegvisomant successfully decreased expression of N-cadherin in the presence of GH, it was unable to decrease the Zeb1 expression level. When exposed to cisplatin, GH increased the expression of Snail, Twist1, and MMP14, but no changes were observed in the expression of E-cadherin, N-cadherin, Zeb1, Slug, TIMP1, or TIMP2 with the addition of GH to cisplatin. The addition of Pegvisomant to cisplatin in the absence of GH increased expression of E-cadherin, TIMP1, and TIMP2 while decreasing expression of N-cadherin, Zeb1, Snail, Slug, Twist1, and MMP14. While co-administration of GH and Pegvisomant in the presence of cisplatin was able to successfully increase expression of E-cadherin and decrease that of N-cadherin, Zeb1, Snail, Twist1, and MMP14, it was unable to increase expression of TIMP1 and TIMP2 or decrease expression of Slug and Twist1. No significant changes in MMP9 expression were observed in the presence of cisplatin. When exposed to gemcitabine, GH increased expression of Slug and Twist1, but no changes in expression were observed in E-cadherin, N-cadherin, Zeb1, MMP9, or TIMP1. The addition of Pegvisomant alone to gemcitabine significantly increased expression of E-cadherin and TIMP1, while decreasing expression of N-cadherin, Zeb1, Slug, Twist1, MMP9, and MMP14. In the presence of GH and gemcitabine, Pegvisomant was still able to successfully increase expression of E-cadherin and TIMP1 and decrease expression of N-cadherin, but was not able to decrease expression of Zeb1, Slug, Twist1, or MMP14. No significant changes in expression of Snail or TIMP2 were observed in the presence of gemcitabine. Additionally, expression levels of Vimentin, TGF-β, and MMP2 were unaffected by all treatment conditions (Figure 8). Taken together, these findings suggest that GH promotes EMT in mouse UC cells, particularly in the presence of chemotherapy, while Pegvisomant inhibits many of these effects, though some markers remain unaffected.

## 3. Discussion

This study integrated transcriptomic analysis and in vitro assays to investigate the role of GH signaling in UC. We found that *GHR* expression correlated with advanced disease stage and reduced survival, despite being lower in UC tumors than in noncancerous bladder tissue. This pattern is consistent in many cancers where targeting GH action has shown therapeutic promise, including melanoma [20,21,22,24,33], pancreatic cancer [23,34,94], breast cancer [13,15,19,26,35,95,96,97,98], and prostate cancer [99,100,101,102,103,104], among others. In these types of cancers, GH plays a key role in creating a tumor microenvironment conducive to cancer progression, despite the fact that GHR is not overexpressed. Notably, GH promoted drug resistance, cellular migration, invasion, and expression of EMT and ECM remodeling markers in vitro, which was reversed with the addition of Pegvisomant.

While endocrine GH secretion by the anterior pituitary gland decreases with age through the process of somatopause [105], peripheral GH secretion, including that by tumor cells, does not, and can even increase [106,107]. Additionally, GHR is expressed by many types of cells in the tumor microenvironment, including tumor cells [108], cancer-associated adipocytes, cancer-associated fibroblasts, immune cells, stromal cells [29], and endothelial cells [109]. This enables highly concentrated, locally produced GH to drive tumor-supportive actions [29,109,110,111,112]. The intricate crosstalk between GH action and the tumor microenvironment underscores the importance of exploring the relationship between *GHR* expression and patient survival in UC.

GH action is associated with poor prognosis in terms of overall survival and disease-free survival in many cancers, including breast cancer [15,113], liver cancer [16,114,115], colorectal cancer [116], and gastric cancer [117], among others [19]. However, this relationship has never been explored in the context of UC. Despite the evidence implicating GH in promoting tumor progression and therapy resistance in some cancers, other studies suggest that GH itself may not directly cause cancer. Clinical data from patients receiving GH replacement therapy for GH deficiency have not consistently demonstrated an increased risk of cancer development [118,119,120]. Long-term follow-up with patients with acromegaly, characterized by chronic elevation of circulating GH and IGF-1, have reported an association with certain cancers, but this remains controversial, as confounding factors such as age, comorbidities, and genetic predispositions may be causative, rather than increased GH and IGF-1 [5,121,122]. Overall, while GH may contribute to tumor progression through its effects on proliferation, survival, and therapy resistance, current evidence does not definitively support the notion that GH alone is a primary driver of cancer development.

Sex- and stage-specific analyses revealed further complexity. *GHR* and *IGF1* were inversely correlated with survival in male and combined cohorts, particularly at later stages. This could potentially be due to the fact that sex hormones regulate transcription of *GH1* differently [123,124,125]. Additionally, female patients represented only approximately one-third of male patients at each clinical stage, a disparity that reflects the greater prevalence of UC in males and may influence the observed data trends. Interestingly, *GH1* displayed opposite correlations depending on tumor stage and patient sex. In addition to the pronounced gender disparity in UC prevalence, there is also a significant racial disparity. UC is most frequently diagnosed in Caucasians, where tumoral *GHR* and *IGF1* were inversely correlated with survival. Although UC is less prevalent in Black individuals, they experience disproportionately higher mortality [126,127]. In our study, *IGF1R* expression was inversely correlated with survival in Black patients, and *GHR* showed a similar trend that did not reach statistical significance, which may be one of many factors that contribute to their increased mortality rate. Notably, in Asian patients, who have the highest global UC prevalence [128] we observed a positive correlation between *GHR* expression and survival. These findings highlight the complex interplay between GH signaling and demographic factors in UC, emphasizing the importance of population-specific analyses.

While tumoral *GH1* expression can provide some insight into GH action, it may not be as reliable as an indicator of active GH signaling as *GHR* expression, since *GHR* levels more directly reflect a tumor’s capacity to respond to circulating GH and activate downstream signaling pathways. Additionally, IGF-1 and its receptor play a critical role in mediating GH signaling and exertion of mitogenic and anti-apoptotic effects in cancer. Given that *IGF1* and *IGF1R* can also be expressed by tumor cells and influence oncogenic processes both in GH-dependent and GH-independent manners [129], their expression levels may further complicate the assessment of the activity of the GH pathway in UC. Therefore, *GHR* expression, along with *IGF1* and *IGF1R* levels, may provide a more comprehensive understanding of GH signaling in tumor progression.

Beyond its impact on tumor progression and survival, GH action is also a well-established driver of resistance to cancer therapies. An extensive body of research has emerged over the past decade eliciting the molecular mechanisms by which GH influences this process, including but not limited to active drug efflux and cellular detoxification, promotion of EMT and ECM remodeling, and evasion of apoptosis [130]. Additionally, the primary hurdle to overcome in treating UC is that of resistance to therapy [42,43,44,45,47].

Among these mechanisms, the regulation of active drug efflux through the modulation of ABC transporters plays a central role in reducing the intracellular concentration, and thereby efficacy, of chemotherapeutics. ABC transporters are commonly upregulated across cancer types [19,131,132], a phenomenon further exacerbated by both chemotherapy and GH, but reversible by inhibiting GH action [20,22,33]. GH, directly or indirectly via IGF-1, has been shown to increase expression of the ABCB, ABCC, and ABCG subfamilies of ABC transporters in breast cancer [15], melanoma [20,33], ovarian cancer [133,134], colorectal cancer [135], and leukemia [136]. In UC, ABCG2 mediates doxorubicin resistance [137], while lower expression of ABCB1, ABCC2, and ABCG2 correlates with better response to enfortumab vedotin [90]. In our study, tumoral *GHR* expression correlated positively with multiple ABC transporters (e.g., *ABCB1*, *ABCB4, ABCB5*, *ABCC8*, *ABCC9*, and *ABCG4*) in both sexes and with additional transporters (*ABCB10*, *ABCC6*, and *ABCG2*) in male patients. In vitro, GH increased ABCB1 and ABCC2 expression in mouse UC cells treated with cisplatin and gemcitabine, an effect reversed by Pegvisomant. Pegvisomant also reduced ABCC1 expression in the presence of cisplatin. Emerging evidence supports a role for the ABCA subfamily in chemoresistance [138,139,140], with GH upregulating ABCA6 and ABCA8 in pancreatic cancer [23]. Consistently, we observed positive correlations between *GHR* and *ABCA8* and *ABCA9* in both sexes, *ABCA13* in females, and *ABCA6* in males. These findings reinforce GH’s role in ABC-transporter-mediated therapy resistance and highlight Pegvisomant’s potential to counteract this pathway in UC.

In addition to its regulation of ABC transporters, GH also influences drug metabolism through regulation of cytochrome P450 enzymes [141,142,143], which are responsible for metabolizing the majority of clinical drugs, including chemotherapeutics [87]. It is important to recognize that the metabolism of drugs reduces their therapeutic efficacy [144]. In this study, *GHR* expression correlated with several CYPs (*CYP1B1*, *CYP2A6*, *CYP2A7*, *CYP2B7P1*, *CYP2J2*, and *CYP2U1*) in both male and female patients with UC, suggesting that GH may contribute to altered drug metabolism and reduced chemotherapy efficacy in UC via CYP regulation.

In addition to modulating drug metabolism, GH plays a key role in metastatic progression through regulation of EMT and ECM remodeling [30]. EMT, often described as a “phenotype switch” that signals the onset of metastasis, is more accurately viewed as a sliding scale rather than an abrupt switch. This process involves the loss of epithelial characteristics and acquisition of invasive, mesenchymal traits [78]. In this study, GH significantly enhanced migration and invasion in both human and mouse UC cells, effects that were reversed by Pegvisomant. While EMT is essential in development, it becomes dysregulated in cancer [145], and GH has been shown to induce EMT in both normal and cancerous tissues [19,24,25,27,130,146]. Our transcriptomic analyses identified strong positive correlations between tumoral *GHR* expression and numerous EMT-related genes (e.g., *ZEB1/2*, *SNAI1*, *TWIST ½*, *CDH2*, *FN1*, and *TGFB* isoforms) in both sexes. In mouse UC cells, GH upregulated N-cadherin, Snail, Slug, and Twist1 in the presence of chemotherapy, while Pegvisomant restored E-cadherin and reduced N-cadherin, Snail, Slug, Zeb1, and Twist1 expression. In the absence of chemotherapy, GH selectively increased N-cadherin and Zeb1, with Pegvisomant reversing N-cadherin upregulation. Vimentin levels were unchanged, suggesting its regulation may be independent of GH signaling in UC. Taken together, these findings support a role for GH in promoting EMT and metastasis, highlighting the potential of Pegvisomant to counteract these processes.

ECM remodeling is a key component of metastasis and is strongly influenced by GH, largely through regulation of MMPs, which digest ECM and promote tumor invasion [24,27,59,147] GH has been shown to upregulate MMP2 and MMP9 in multiple cancers [24,31,32,93], and, in UC, elevated circulating levels of these MMPs are linked to aggressive disease and may serve as biomarkers [148,149,150,151,152]. In this study, *GHR* expression correlated with several MMPs, including *MMP2*, *MMP3*, *MMP 11*, and *MMP16*, in both sexes and additional MMPs in male patients. GH increased expression of MMP14 in cisplatin-treated cells, an effect reversed by Pegvisomant, which also reduced expression of MMP9 and MMP14 and increased expression of TIMP1 and TIMP2 in a chemotherapy-dependent manner. In the absence of chemotherapy, no significant changes were observed, and MMP2 and TGF-β expression remained unchanged across all treatment conditions, suggesting selective GH regulation under treatment stress. GH also promotes collagen synthesis and fibrosis, processes known to support tumor progression [153]. All significant correlations between tumoral *GHR* and collagen genes were positive, reinforcing the role of GH in ECM remodeling and metastatic potential in UC.

Beyond its role in metastasis, GH promotes tumor cell survival and therapy resistance by inhibiting apoptosis [154,155,156]. The mechanism by which GH exerts this action is via upregulation of anti-apoptotic genes, such as BCL2, and downregulation of pro-apoptotic genes, such as p53 and caspases [30]. In this study, tumoral *GHR* correlated positively with several anti-apoptotic genes (*NFIL3*, *BCL2*, *TNFRSF11B*, and *TSFRSF10D*), with additional associations observed in a sex-specific manner. Conversely, *GHR* was negatively correlated with key pro-apoptotic genes, including *AIMP1* and *DIABLO* in both sexes, *TRADD* in female patients, and *CASP6*, *CASP8*, *CASP9*, *BAK1*, and *FADD* in male patients. These findings support the role of GH in fostering a pro-survival TME, thereby reducing the effectiveness of anticancer therapies.

Despite providing valuable insight into the potential role of GH in UC, several limitations should be noted. First, the in silico analyses rely solely on transcriptomic data, which may not reflect protein expression due to post-transcriptional regulation [157]. We sought to address this in part through in vitro validation, but did not perform functional studies to confirm causality. In vivo studies are necessary to determine whether GH directly drives therapy resistance and tumor progression. Additionally, this work focuses on tumor-intrinsic *GHR* expression and does not account for the complex interactions within the tumor microenvironment, including crosstalk with stromal cells, immune components, and ECM interactions. Since GH signaling is known to affect multiple cell types, a more comprehensive approach incorporating these factors could provide a clearer mechanistic understanding.

Collectively, our findings suggest a potential role for GH action in UC progression, survival outcomes, and resistance to therapy by enhancing drug efflux, EMT, and ECM remodeling. The observed inverse correlation between *GHR* expression and survival, particularly in advanced clinical stages, supports the notion that GH signaling may contribute to aggressive disease phenotypes. Additionally, the differential expression patterns of *GH1*, *IGF1*, and *IGF1R* across sex and racial groups underscore the complexity of GH action in UC and its potential as a prognostic biomarker. Importantly, Pegvisomant has the ability to reverse GH-driven oncogenic processes, even in the absence of exogenous GH, indicating its ability to block both endocrine and paracrine/autocrine GH signaling. These findings emphasize the need for further investigation into the therapeutic targeting of GH action in UC, which may provide novel strategies to improve patient outcomes, particularly in advanced disease stages. Given the significant overlap between GH signaling and key pathways in cancer progression, future studies should focus on validating these findings in vivo to further assess the feasibility of targeting GH action in UC treatment [44,45,46,47].

## 4. Materials and Methods

### 4.1. GHR Expression Analysis

*GHR* mRNA transcript-level comparison between normal and tumoral tissues of patients with bladder cancer in TCGA database was performed using Gene Expression Profiling Interactive Analysis 2 (GEPIA2) web tool (http://gepia2.cancer-pku.cn/#index; accessed 27 May 2025) [158]. Expression of *GHR* in different clinical grades and stages of patients with bladder cancer in TCGA database was evaluated using Tumor-Immune System Interaction DataBase (TISIDB) web tool (http://cis.hku.hk/TISIDB/index.php; accessed 27 May 2025) [159].

### 4.2. Survival Curves

Transcriptomic data archived in TCGA database obtained from patients with bladder cancer were used to perform a multivariate analysis to generate Kaplan–Meier plots for overall survival (OS) with the KM plotter web tool (https://kmplot.com/analysis/; accessed 27 May 2025). With *GHR*, *GH1*, *IGF1R*, or *IGF1* as the reference gene, sex-based (all, female, male) stage-based, combinations of sex- and stage-based, and race-based (Asian, Black, white) correlations with patient survival were obtained and OS plots were generated for a total of 405 patients with bladder cancer [160]. OS and disease-free survival (DFS) curves were also generated for 394 patients with bladder cancer in TCGA database with *GHR* as the reference gene and patients sorted at the median *GHR* mRNA expression level using GEPIA2 web tool [158].

### 4.3. Gene Expression Correlation Analyses

Spearman correlation analyses of differentially expressed mRNAs with respect to *GHR* mRNA expression in tumor samples of patients with bladder cancer in TCGA database were performed using LinkedOmics web tool (http://linkedomics.zhang-lab.org/login.php; accessed 27 May 2025) [161]. The corresponding correlation coefficients, respective *p* values, and false discovery rates (FDRs) were used to create heat maps in Microsoft Excel. In TCGA dataset, transcriptomic data were available for 108 female and 304 male patients with bladder cancer.

### 4.4. Cell Culture

One human and one mouse cell line were selected for our studies. UM-UC-3 (human UC; purchased from ATCC #CRL-1749, Manassas, VA, USA) and MB49 (C57BL/6-background murine UC; purchased from Sigma-Aldrich #SCC148, St. Louis, MO, USA) were maintained in EMEM (ThermoFisher #11095098, Waltham, MA, USA) and DMEM growth media (ThermoFisher #11995040), respectively, supplemented with 10% fetal bovine serum (FBS; ThermoFisher #10082147) and 1% penicillin-streptomycin (ThermoFisher #15140122), referred to hereafter as complete growth medium, at 37 °C and 5% CO_2_. UM-UC-3 is an extremely commonly used cell line to model high-grade UC with nearly 900 citations as of 18 February 2025. Additionally, UM-UC-3 has the highest level of GHR expression among the 26 commercially available cell lines (Figure S5) (proteinatlas.org; accessed 27 May 2025; [162]). We chose MB49 for our studies as we wanted to use a mouse UC cell line that is compatible with C57BL/6J-background mice, and it is the only commercially available one.

### 4.5. GH, Pegvisomant, and Chemotherapy Treatments

Recombinant active human (R&D Systems #1067-GH, Minneapolis, MN, USA) and bovine (ProSpecBio #CYT-636, Rehovot, HaMerkaz, Israel) GH were administered to human cells at 50 ng/mL and murine cells at 200 ng/mL, respectively. Pegvisomant (Pfizer, New York, NY, USA) was administered to cells at 13,500 ng/mL. Chemotherapies (gemcitabine (Selleck Chemicals #S1714, Houston, TX, USA) and cisplatin (Selleck Chemicals #S1166)) were administered to cells at sub-EC_50_ concentrations so as not to significantly reduce cell viability. All treatments were performed in growth medium containing 2% FBS or serum-free medium for Western blots of GH signaling intermediates.

### 4.6. EC_50_ Determination

Cells were seeded at 50,000 cells/well in 96-well plates in complete growth medium. After 24 h, 10 serial dilutions of chemotherapy drugs made in growth medium supplemented with 2% FBS and 1% penicillin-streptomycin were added to cells and incubated for 48 h at 37 °C. After 48 h, drug dilutions were removed, and cells were incubated with 0.0045% resazurin (ThermoFisher #R12204) solution until solutions in control wells reduced to resorufin and appeared bright pink. Absorbance was read at 570 nm with a reference wavelength of 600 nm. The EC_50_ values were calculated using Prism 10 software (Figure S6; GraphPad, San Diego, CA, USA). Subsequent chemotherapy treatments were performed at drug concentrations that were 10% lower than their respective EC_50_ values so as not to significantly reduce cell viability. Using sub-EC_50_ concentrations of chemotherapies also allowed us to demonstrate that they are efficacious at lower concentrations in the presence of the GHR antagonist Pegvisomant.

### 4.7. Real-Time Quantitative PCR (RT-qPCR)

Total RNA was isolated from cultured cells using a commercially available kit (IBI Scientific #IB4730, Dubuque, IA, USA). Reverse-transcription PCR was performed using a commercially available kit (ThermoFisher #4368814). cDNA was quantified using NanoDrop 2000 (ThermoFisher #ND-2000) and diluted to 200 ng/µL in nuclease-free water. We used 500 ng of cDNA with gene- and species-specific forward and reverse primers at 10 µM (Table 1; all manufactured by Sigma-Aldrich, St. Louis, MO, USA) and SYBR green/ROX qPCR mix (ThermoFisher #K0222) to amplify specified genes using the QuantStudio 3 qPCR machine and software (ThermoFisher #A28567). RNA expression was first normalized against two reference genes (*ACTB* and *GAPDH* in human cells; *Actb* and *Tubb5* in mouse cells), and expression levels were then quantified using the 2^−∆∆Ct^ method in Microsoft Excel.

### 4.8. Western Blot

Total protein was isolated from cells after 12 min of treatment for GH signaling intermediates, as the phosphorylation levels reach a peak between 5 and 30 min following stimulation with GH, or 48 h otherwise, as this incubation time allows for a significant change in protein levels to occur after a stimulus, using RIPA buffer (ThermoFisher #J62524.AE) diluted to 1.5× and supplemented with 1% PMSF (ThermoFisher #36978) and 1% protease-phosphatase inhibitor (ThermoFisher #78442). Protein concentration was determined using the Bradford assay (BioRad #5000006, Hercules, CA, USA). A total of 80 µg of protein for GH signaling intermediates or 20 µg otherwise was boiled with Laemmli buffer (BioRad #1610747) for 8 min before separation by SDS-PAGE. Proteins were transferred to a PVDF membrane overnight at 4 °C. The membrane was blocked in 5% nonfat dry milk in tris-buffered saline with Tween-20 (TBS/T) for 1 h at room temperature before overnight incubation with primary antibody diluted in blocking buffer at 4 °C (Table 2; Cell Signaling Technology (CST), Danvers, MA, USA; ProteinTech, Rosemont, IL, USA; Invitrogen, Waltham, MA, USA; Bioss USA, Woburn, MA, USA). The membrane was then washed with TBS/T, incubated with secondary antibody (CST #7074 1:2000) for 60 min at room temperature, and washed again before application of West Femto Super Signal Chemiluminescence detection reagent (ThermoFisher #34095) and imaging using the Odyssey XF (LI-COR Biosciences, Lincoln, NE, USA) or Azure 300 (Azure Biosystems, Dublin, CA, USA) imaging system. Densitometry analysis was performed using ImageJ software (version 1.51) [163]. Protein expression was normalized to a loading control (β-actin) before analysis.

### 4.9. Migration Assay

Cells were seeded at 7 × 10^5^ cells/mL in complete growth medium inside each well of 2-well culture inserts (Ibidi #80209, Fitchburg, WI, USA) inserted into 12-well plates. After 24 h, culture inserts were removed, cells were washed with 1X sterile PBS, and culture medium supplemented with 2% FBS and 1% penicillin-streptomycin was added to cells. Images were captured using a Cytation 3 Imaging Reader (BioTek Instruments, Winooski, VT, USA) and Gen5 software (BioTek Instruments). Culture medium was removed, GH and Pegvisomant treatments were administered, and cells were incubated at 37 °C supplemented with 5% CO_2_ for 24 h. After 24 h, images were captured again and compared to original images using ImageJ software [163] and a software plugin that uses a computer vision segmentation algorithm to provide an unbiased method to quantify the cell-free region created by the culture insert, correct for any nonzero angle, and assess the average width along with variations [164].

### 4.10. Invasion Assay

Cells were seeded in 25 cm^2^ culture flasks in complete growth medium and pre-treated for 1 week with GH and Pegvisomant once every 48 h in growth medium containing 2% FBS. Cells were then enzymatically removed from the culture flasks using 0.25% trypsin-EDTA before being seeded at 0.5 × 10^5^ (MB49) or 1.0 × 10^5^ cells/mL (UM-UC-3) in the basement membrane chamber of the invasion assay kit (Cell BioLabs #CBA-112, San Diego, CA, USA) with GH and Pegvisomant treatments in appropriate suspensions. The basement membrane chambers of the invasion assay kit were then carefully placed into the feeder tray of the invasion assay kit, which contained complete culture medium. The full invasion assay plate, which contained both the basement membrane chamber and the feeder tray, was incubated at 37 °C supplemented with 5% CO_2_ for 24 h. A total of 30 min prior to the end of the 24 h incubation period, cell detachment solution was added to the cell harvesting tray of the invasion assay kit and incubated for 30 min at 37 °C supplemented with 5% CO_2_. At the end of the 24 h incubation period, cells that had invaded through the basement membrane chamber of the invasion assay plate were dislodged by placing the basement membrane chamber onto the cell harvesting tray and tilting it several times. Lysis buffer containing fluorescent dye was added to all samples before incubation for 20 min at room temperature. After 20 min, the mixture from the cell harvesting tray was transferred to a black-walled 96-well plate and fluorescence was measured at 480 nm/520 nm using a Cytation 3 Imaging Reader (BioTek Instruments) and Gen5 software (BioTek Instruments).

### 4.11. Statistical Analyses

All experiments were repeated at least thrice. Tests of variance and homogeneity were performed, followed by parametric (Student’s *t*-test, ANOVA) or non-parametric (Wilcoxon sign-rank test) analyses as appropriate to identify significant differences. Protein expression was normalized to a loading control before analysis. All analyses were performed using Prism 10 software (GraphPad) unless otherwise noted, with significance being defined as *p* ≤ 0.05.

## 5. Conclusions

Together, this work was directed at testing the hypothesis that inhibiting GH action will reverse therapy resistance and improve disease prognosis in UC. This study investigated the role of GHR expression in UC using transcriptomic data from TCGA. We found that GHR expression is associated with advanced UC stages, reduced survival, and increased resistance to therapy, indicating its potential as a prognostic biomarker and therapeutic target. Additionally, we sought to confirm these findings in vitro. We observed that GH plays a direct role in promoting therapy resistance and metastatic processes in UC by upregulating ABC transporters, inducing EMT, and modulating ECM remodeling. Pegvisomant effectively counteracts these effects, highlighting its potential as a therapeutic strategy to enhance treatment efficacy in UC.

## Figures and Tables

**Table 1:** Primer sequences (5′ → 3′).

Target Gene	Species	Forward Sequence	Reverse Sequence
*ACTB*	Human	GACGACATGGAGAAAATCTG	ATGATCTGGGTCATCTTCTC
*GAPDH*	Human	CTTTTGCGTCGCCAG	TTGATGGCAACAATATCCAC
*GH1*	Human	AGGAAACACAACAGAAATCC	TTAGGAGGTCATAGACGTTG
*GHR*	Human	CTCCTCAAGGAAGGAAAATTAG	GTGGAATTCGGGTTTATAGC
*IGF1*	Human	TTATTTCAACAAGCCCACAG	AATGTACTTCCTTCTGGGTC
*IGF1R*	Human	AGGGAATTACTCCTTCTACG	TTTATGTCCCCTTTGCTTTG
*IGFBP3*	Human	AATCATCATCAAGAAAGGGC	GAACTTCAGGTGATTCAGTG
*PRL*	Human	GGTTCATCCTGAAACCAAAG	CTTCAGGAGCTTGAGATAATTG
*INS*	Human	CCATCAAGCAGATCACTG	CACTAGGTAGAGAGCTTCC
*INSR*	Human	GGAACTACTCCTTCTATGCC	CCTGAAACTTCTTCCATCTTG
*Actb*	Mouse	GATGTATGAAGGCTTTGGTC	TGTGCACTTTTATTGGTCTC
*Tubb5*	Mouse	CTTGTTCGGTACCTACATTG	CATGTTCATCGCTTATCACC
*Gh1*	Mouse	TCCAGTCTGTTTTCTAATGC	TCGAACTCTTTGTAGGTGTC
*Ghr*	Mouse	ACTGTCCAGTGTACTCATTG	CTGGATATCTTCTTCACATGC
*Igf1*	Mouse	GACAAACAAGAAAACGAAGC	ATTTGGTAGGTGTTTCGATG
*Igf1r*	Mouse	AGAACCGAATCATCATAACG	TTTTAAATGGTGCCTCCTTG
*Igfbp3*	Mouse	CTGAATCATCTGAAGTTCCTC	GGCACTGCTTCTTCTTATAG
*Ins*	Mouse	AGCAGGAAGGTTATTGTTTC	ACATGGGTGTGTAGAAGAAG
*Insr*	Mouse	CAAACAGATGCCACTAATCC	CTTTGAGACAATAATCCAGCTC

**Table 2:** Primary antibody information.

Target	Dilution Used	Target Species	Manufacturer	Catalog Number
β-actin	1:3000	H/M	CST	4970
Phospho-STAT5a/b	1:1000	H/M	R&D Systems	MAB41901
Phospho-STAT3	1:500	H/M	CST	9145
Phospho-p44/42 MAPK	1:2000	H/M	CST	9101
Phospho-Akt	1:1000	H/M	CST	9271
Phospho-Src	1:500	H/M	CST	2101
STAT5	1:1000	H/M	CST	25656
STAT3	1:1000	H/M	CST	12640
p44/42 MAPK	1:2000	H/M	CST	9102
Akt	1:1000	H/M	CST	4691
Src	1:1000	H/M	CST	2109
E-cadherin (CDH1)	1:10,000	H/M	ProteinTech	20874-1-AP
N-cadherin (CDH2)	1:1000	H/M	CST	13116
Vimentin	1:1000	H/M	CST	5741
ZEB1	1:1000	H	CST	3396
	1:1000	M	ProteinTech	21544-1-AP
Snail (SNAI1)	1:500	H	CST	3879
	1:1000	M	ProteinTech	13099-1-AP
Slug (SNAI2)	1:500	M	CST	9585
	1:10,000	H	ProteinTech	12129-1-AP
TWIST1	1:500	H	CST	90445
	1:2000	M	ProteinTech	25465-1-AP
MMP2	1:1000	H	CST	87809
	1:1000	M	ProteinTech	10373-2-AP
MMP9	1:1000	H	CST	13667
	1:1000	M	ProteinTech	10375-2-AP
MMP14	1:1000	H/M	Invitrogen	MA5-32076
TIMP1	1:1000	M	CST	63363
	1:500	H	ProteinTech	16644-1-AP
TIMP2	1:1000	H/M	CST	5738
TGF-β	1:500	H/M	CST	3711
ABCB1	1:1000	H/M	ProteinTech	22336-1-AP
ABCC1	1:500	H	CST	72202
	1:1000	M	Abcam	AB260038
ABCC2	1:500	H	CST	12559
	1:1000	M	Invitrogen	PA5-86719
ABCG2	1:2000	H/M	ProteinTech	27286-1-AP
GHR	1:1000	H/M	Bioss USA	bs-0654R

## Data Availability

All the data described in the manuscript are contained in the main text or supporting information. Additional requests are to be addressed to the corresponding author.

## Supplementary Materials

The following supporting information can be downloaded at: https://www.mdpi.com/article/10.3390/ijms26157113/s1.

## Abbreviations

The following abbreviations are used in this manuscript:

GH	Growth hormone
LS	Laron syndrome
GHR	Growth hormone receptor
EMT	Epithelial-to-mesenchymal transition
ECM	Extracellular matrix
ABC	ATP-binding cassette
UC	Urothelial carcinoma
IGF-1	Insulin-like growth factor 1
MMP	Matrix metalloproteinase
TIMP	Tissue inhibitor of metalloproteinase
TCGA	The Cancer Genome Atlas
HR	Hazard ratio
PRL	Prolactin
PRLR	Prolactin receptor
InsR	Insulin receptor
Ins	Insulin
STAT	Signal transducer and activator of transcription
JAK	Janus kinase
MAPK	p42/44 mitogen-activated protein kinase
AKT	Protein kinase B
TGF-β	Transforming growth factor beta
