# Supplementary material for: Growth Hormone Signaling in Bladder Cancer: Transcriptomic Profiling of Patient Samples and In Vitro Evidence of Therapy Resistance via ABC Transporters and EMT Activation
Source: Int J Mol Sci. 2025 Jul 23;26(15):7113. doi: 10.3390/ijms26157113 (PMC12345844; doi:10.3390/ijms26157113)
Supplement: Supplementary file 1 [file ijms-26-07113-s001.zip › ijms-3696387-supplementary.pptx]

## Slide 1
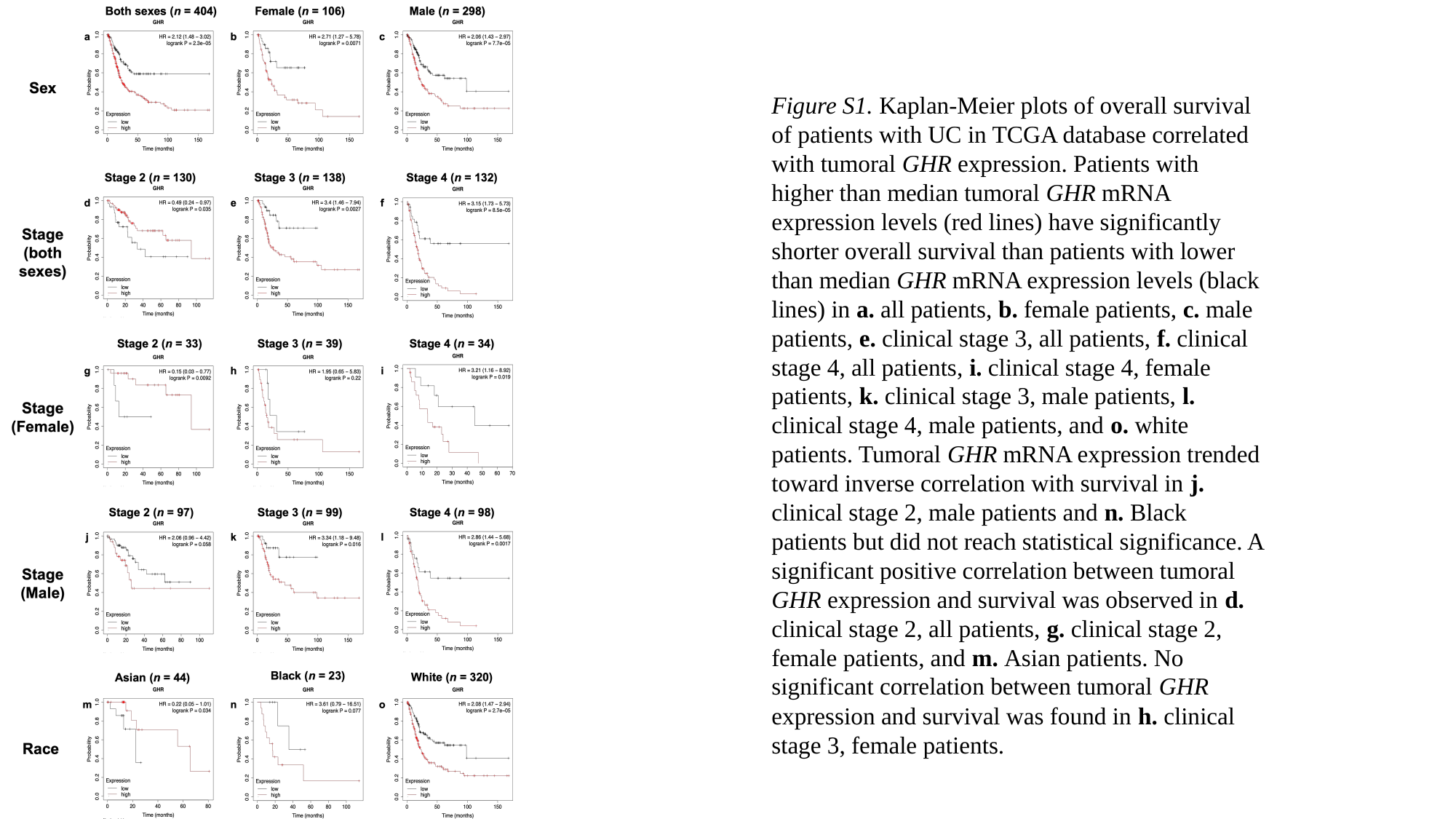

Figure S1. Kaplan-Meier plots of overall survival of patients with UC in TCGA database correlated with tumoral GHR expression. Patients with higher than median tumoral GHR mRNA expression levels (red lines) have significantly shorter overall survival than patients with lower than median GHR mRNA expression levels (black lines) in a. all patients, b. female patients, c. male patients, e. clinical stage 3, all patients, f. clinical stage 4, all patients, i. clinical stage 4, female patients, k. clinical stage 3, male patients, l. clinical stage 4, male patients, and o. white patients. Tumoral GHR mRNA expression trended toward inverse correlation with survival in j. clinical stage 2, male patients and n. Black patients but did not reach statistical significance. A significant positive correlation between tumoral GHR expression and survival was observed in d. clinical stage 2, all patients, g. clinical stage 2, female patients, and m. Asian patients. No significant correlation between tumoral GHR expression and survival was found in h. clinical stage 3, female patients.

## Slide 2
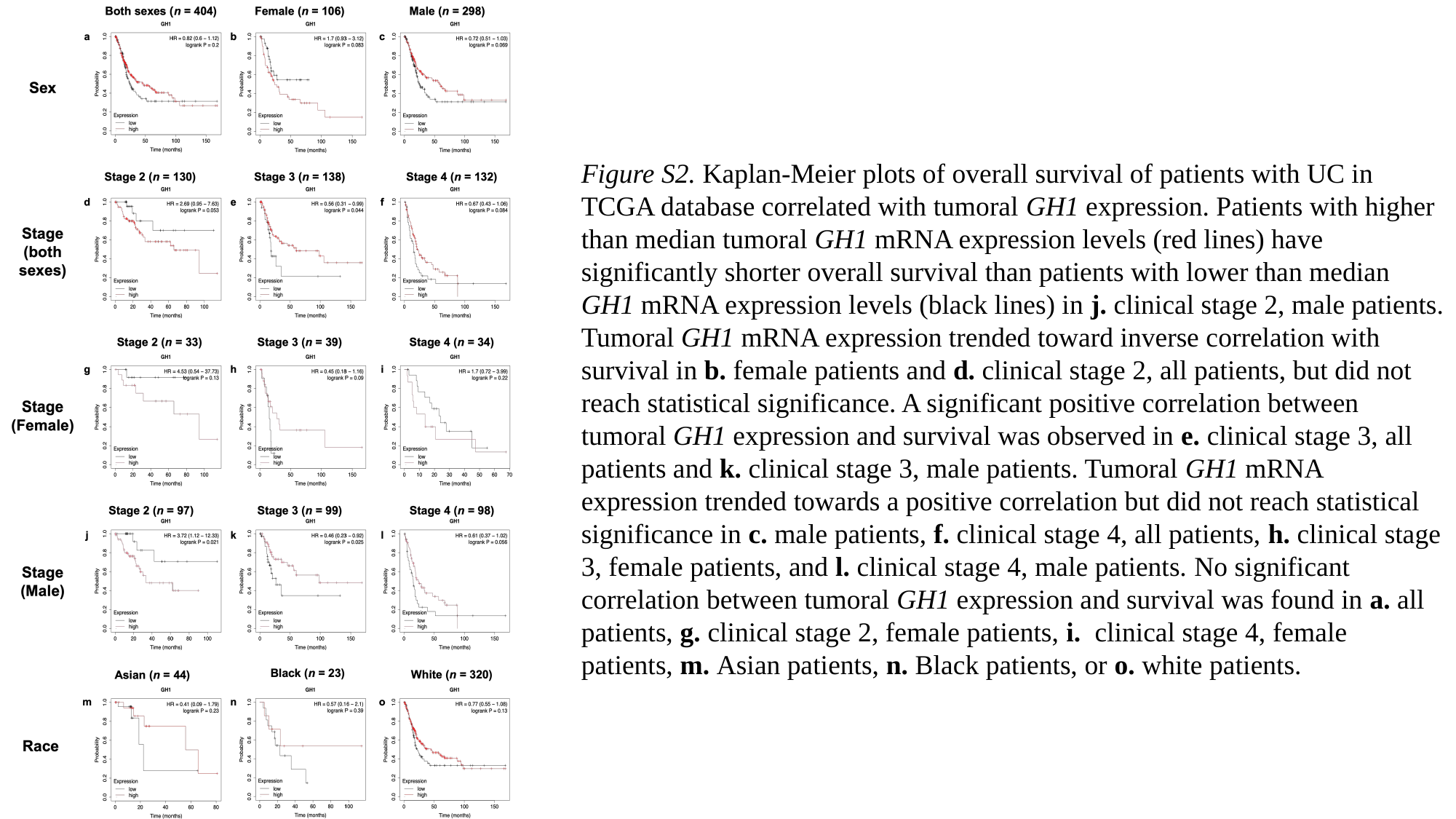

Figure S2. Kaplan-Meier plots of overall survival of patients with UC in TCGA database correlated with tumoral GH1 expression. Patients with higher than median tumoral GH1 mRNA expression levels (red lines) have significantly shorter overall survival than patients with lower than median GH1 mRNA expression levels (black lines) in j. clinical stage 2, male patients. Tumoral GH1 mRNA expression trended toward inverse correlation with survival in b. female patients and d. clinical stage 2, all patients, but did not reach statistical significance. A significant positive correlation between tumoral GH1 expression and survival was observed in e. clinical stage 3, all patients and k. clinical stage 3, male patients. Tumoral GH1 mRNA expression trended towards a positive correlation but did not reach statistical significance in c. male patients, f. clinical stage 4, all patients, h. clinical stage 3, female patients, and l. clinical stage 4, male patients. No significant correlation between tumoral GH1 expression and survival was found in a. all patients, g. clinical stage 2, female patients, i. clinical stage 4, female patients, m. Asian patients, n. Black patients, or o. white patients.

## Slide 3
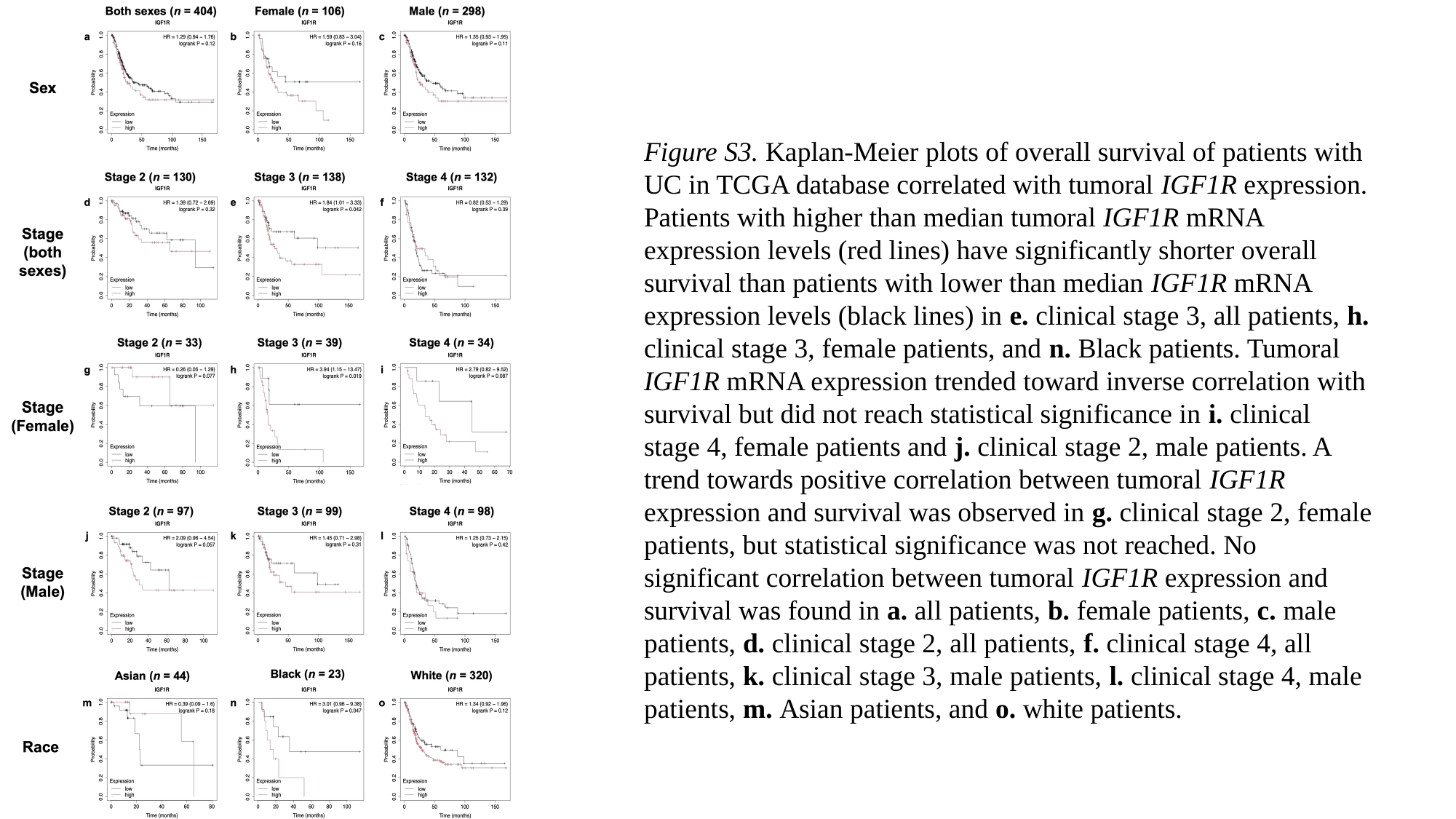

Figure S3. Kaplan-Meier plots of overall survival of patients with UC in TCGA database correlated with tumoral IGF1R expression. Patients with higher than median tumoral IGF1R mRNA expression levels (red lines) have significantly shorter overall survival than patients with lower than median IGF1R mRNA expression levels (black lines) in e. clinical stage 3, all patients, h. clinical stage 3, female patients, and n. Black patients. Tumoral IGF1R mRNA expression trended toward inverse correlation with survival but did not reach statistical significance in i. clinical stage 4, female patients and j. clinical stage 2, male patients. A trend towards positive correlation between tumoral IGF1R expression and survival was observed in g. clinical stage 2, female patients, but statistical significance was not reached. No significant correlation between tumoral IGF1R expression and survival was found in a. all patients, b. female patients, c. male patients, d. clinical stage 2, all patients, f. clinical stage 4, all patients, k. clinical stage 3, male patients, l. clinical stage 4, male patients, m. Asian patients, and o. white patients.

## Slide 4
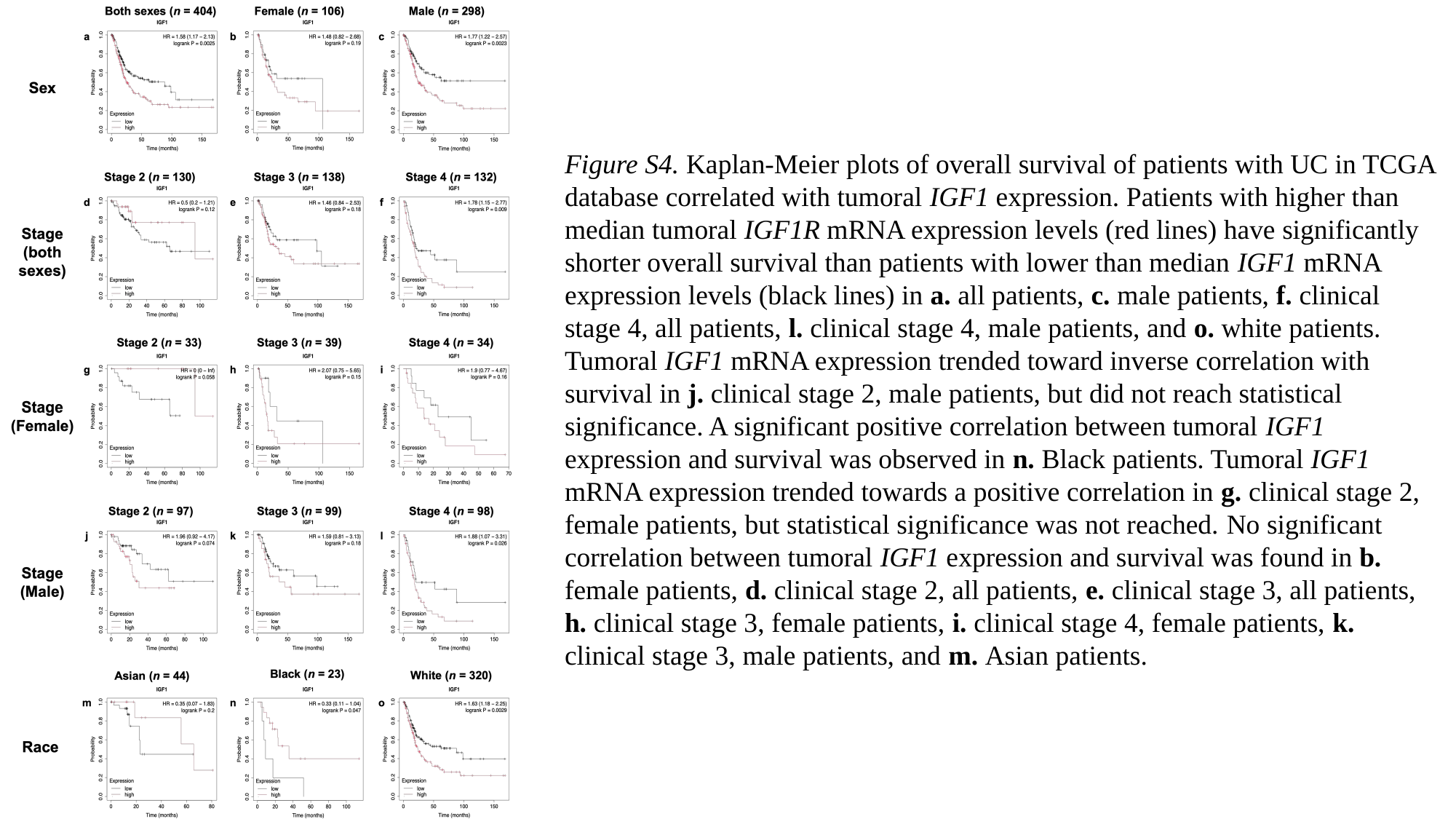

Figure S4. Kaplan-Meier plots of overall survival of patients with UC in TCGA database correlated with tumoral IGF1 expression. Patients with higher than median tumoral IGF1R mRNA expression levels (red lines) have significantly shorter overall survival than patients with lower than median IGF1 mRNA expression levels (black lines) in a. all patients, c. male patients, f. clinical stage 4, all patients, l. clinical stage 4, male patients, and o. white patients. Tumoral IGF1 mRNA expression trended toward inverse correlation with survival in j. clinical stage 2, male patients, but did not reach statistical significance. A significant positive correlation between tumoral IGF1 expression and survival was observed in n. Black patients. Tumoral IGF1 mRNA expression trended towards a positive correlation in g. clinical stage 2, female patients, but statistical significance was not reached. No significant correlation between tumoral IGF1 expression and survival was found in b. female patients, d. clinical stage 2, all patients, e. clinical stage 3, all patients, h. clinical stage 3, female patients, i. clinical stage 4, female patients, k. clinical stage 3, male patients, and m. Asian patients.

## Slide 5
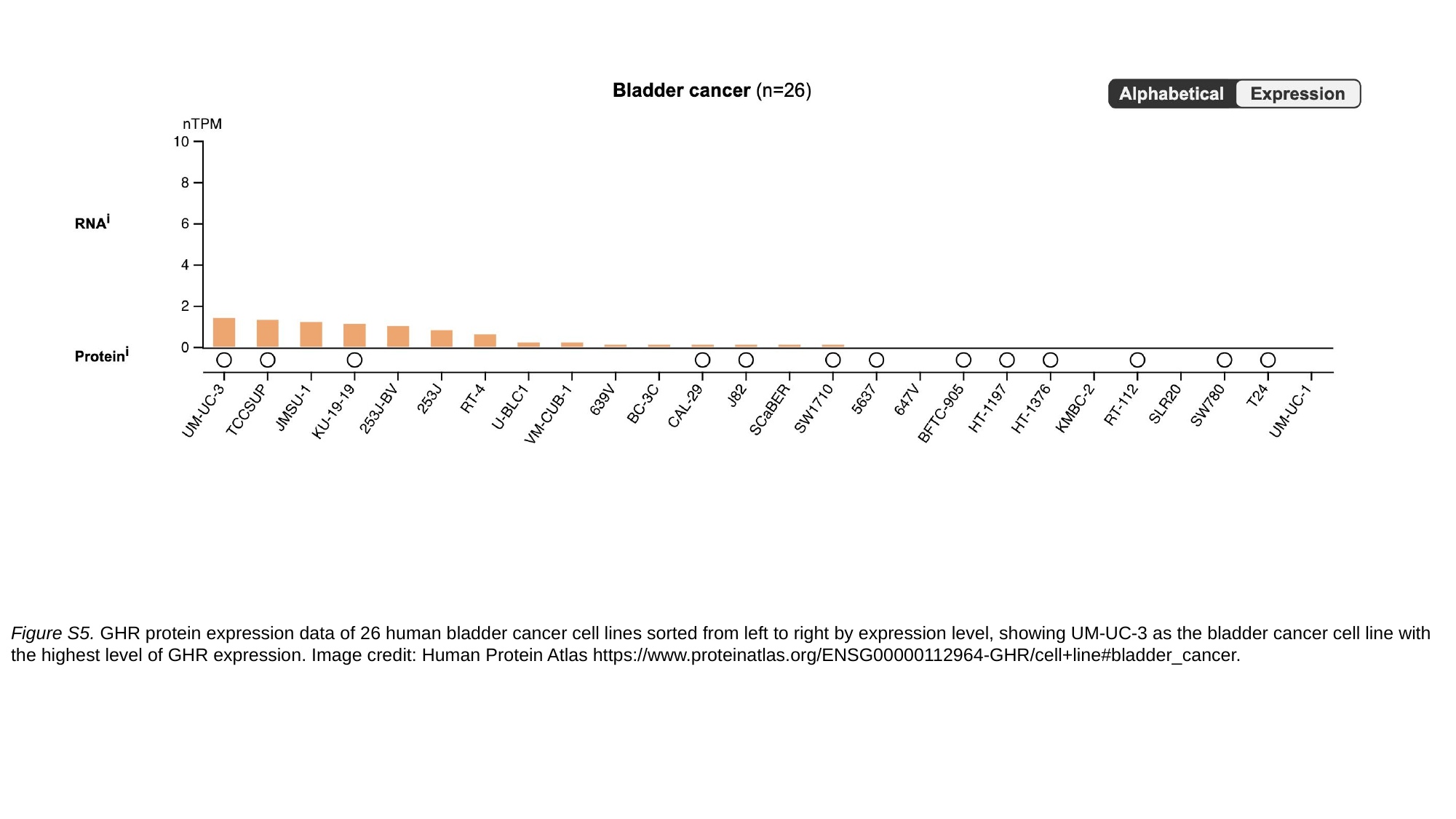

Figure S5. GHR protein expression data of 26 human bladder cancer cell lines sorted from left to right by expression level, showing UM-UC-3 as the bladder cancer cell line with the highest level of GHR expression. Image credit: Human Protein Atlas https://www.proteinatlas.org/ENSG00000112964-GHR/cell+line#bladder_cancer.

## Slide 6
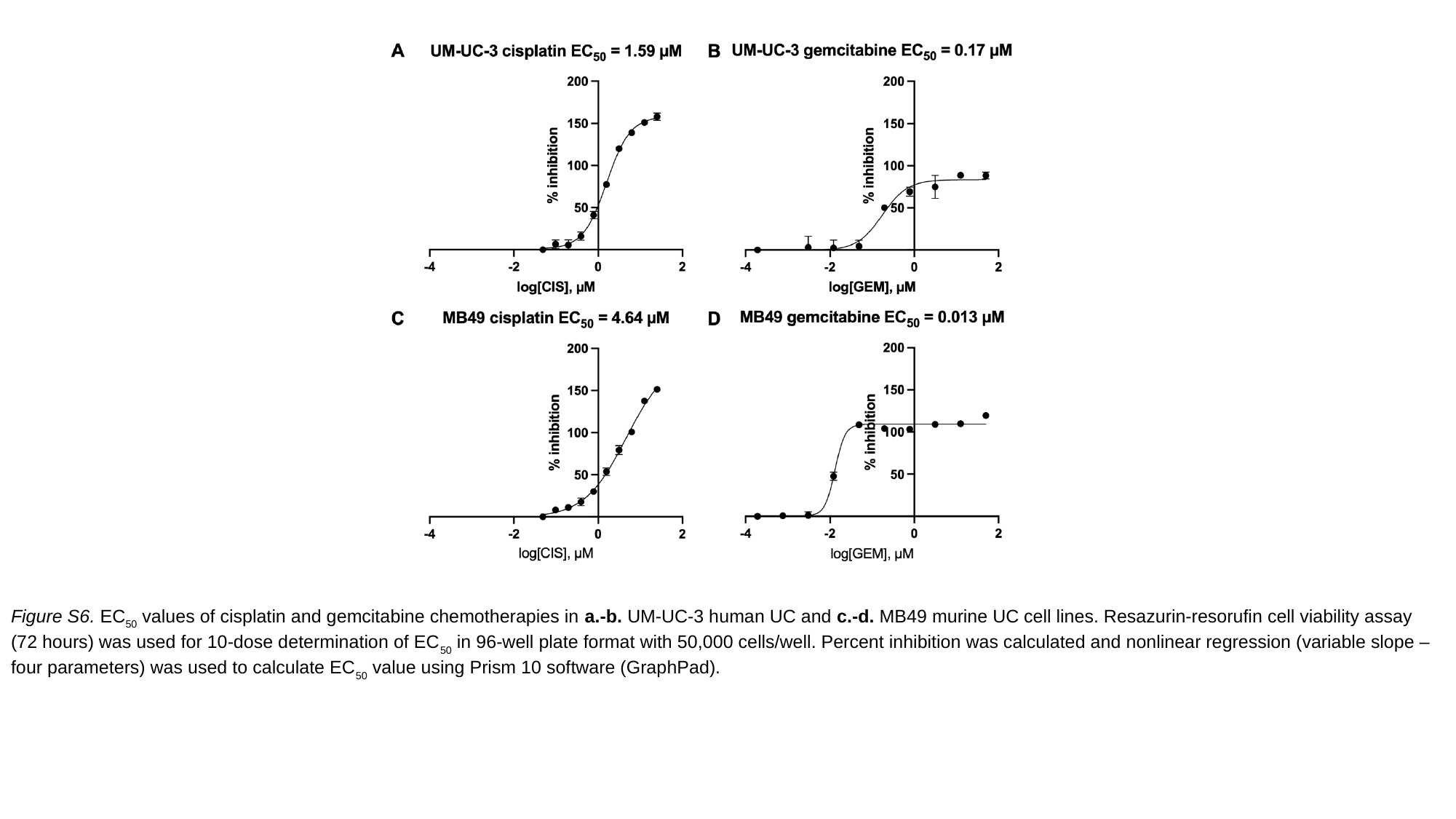

Figure S6. EC50 values of cisplatin and gemcitabine chemotherapies in a.-b. UM-UC-3 human UC and c.-d. MB49 murine UC cell lines. Resazurin-resorufin cell viability assay (72 hours) was used for 10-dose determination of EC50 in 96-well plate format with 50,000 cells/well. Percent inhibition was calculated and nonlinear regression (variable slope – four parameters) was used to calculate EC50 value using Prism 10 software (GraphPad).
